# Renal biochemical and histopathological alterations of diabetic rats under treatment with hydro alcoholic *Morus nigra* extrac

**DOI:** 10.15171/jrip.2017.10

**Published:** 2016-11-22

**Authors:** Mohammad Rahimi-Madiseh, Azar Naimi, Esfandiar Heydarian, Mahmoud Rafieian-Kopaei

**Affiliations:** ^1^Medical Plants Research Center, Shahrekord University of Medical Sciences, Shahrekord, Iran; ^2^Department of Pathology, Isfahan University of Medical Sciences, Isfahan, Iran; ^3^Clinical Biochemistry Research Center, Shahrekord University of Medical Sciences, Shahrekord, Iran

**Keywords:** Kidney, Rat, Diabetes mellitus, *Morus nigra*

## Abstract

**Introduction:** Morus nigra fruit is known to have antioxidant effects and used to control the blood sugar level in traditional medicine.

**Objectives:** This study was conducted to investigate the biochemical and histopathological changes in the serum and kidneys of diabetic rats treated with hydroalcoholic *M. nigra* extract.

**Materials and Methods:** In this study, 60 male Wistar rats were divided into five groups of 12 each. After induction of diabetes with alloxan, the diabetic rats were treated with hydroalcoholic extract of *M. nigra* at different concentrations. Then, the animals were anesthetized and the serum levels of glucose, creatinine, and urea as well as kidney tissue catalase level measured. The kidney tissue was also histopathologically examined.

**Results:** Milder glomerular damage was seen in the group treated with 800 mg/kg of the *M. nigra* extract compared with diabetic and positive controls, and no difference in the expansion of mesenchymal tissue into renal glomerular vessels observed between the group treated with 800 mg/kg of *M. nigra* extract and diabetic and positive controls. Furthermore, creatinine levels were significantly higher and urea levels significantly lower in the group treated with 800 mg/kg of *M. nigra* extract than healthy and positive control groups (*P*<0.05).

**Conclusion:** Administration of *M. nigra* extract at 800 mg/kg can prevent kidney tissue damage in diabetic rats and this fruit seems to be beneficial to patients with diabetes.

**Implication for health policy/practice/research/medical education:**

Administration of M. nigra extract at 800 mg/kg can prevent kidney tissue damage in diabetic rats and this fruit seems to be beneficial to patients with diabetes.

## Introduction

Diabetes mellitus is one of the most common metabolic diseases with an estimated global prevalence of approximately 4.6%. According to the estimates, over 280 million people currently suffer from diabetes and the number of people with diabetes in 2015 will exceed 330 million worldwide (1). With onset of diabetes, the patients are at risk of developing several complications, such as nephropathy, that account for the cause of 70% of them (2).

Currently, to treat diseases, including diabetes, or prevent associated complications, chemical drugs are mainly used. These drugs may cause several side effects and, because of having unpleasant and uncontrollable drug interactions, have occasionally forced healthcare professionals to think of developing more pleasant drugs to manage diabetes (3-7). Nowadays, in the light of the confirmed effects of the medicines used in traditional medicine and the chemical drugs’ side effects, use of complementary and alternative medicine is being increasingly welcome by people worldwide (8-10).

Plant-based drugs have long attracted attention worldwide because of their unique benefits and have been used in most communities and different countries thanks to several advantages including phenolic compounds and their antioxidant role (11-13). *Morus nigra* leaf and fruit are among the plant products that are used, in traditional medicine, to control blood sugar in patients with diabetes (14).

## Objectives

Given the significance of diabetes and associated complications, this study was conducted to investigate the biochemical and histopathological changes in the serum and kidneys of diabetic rats treated with hydroalcoholic *M. nigra* extract.

## Materials and Methods

### Experimental protocol

After preparation of hydroalcoholic *M. nigra* extract and purchase of 60 rats from Pasteur Institute of Tehran, Iran, the rats were assigned to five groups of 12 each consisting of; healthy, diabetic (metformin-administered), and positive control group and two groups treated with *M. nigra* fruit extract (at 400 and 800 mg/kg concentrations). Except for the healthy control group, other groups were diabetized with 120 mg/kg alloxan (Sigma, USA) (15). The positive control group were treated with 150 mg/kg metformin (16), and the two *M. nigra* groups gavaged with *M. nigra* fruit extract for eight weeks. Meanwhile, the healthy and diabetic control groups were gavaged with distilled water.

### Measurement and histopathological procedures

At completion of the treatments, the rats were anesthetized with chloroform and their heart blood samples taken and introduced into non-citrate tubes. After blood taking, the rats’ abdomens were opened, their kidneys taken out, and after being washed with normal saline, one kidney was kept in formalin 10% and the other one in freezer at -70°C. The blood samples were left in the laboratory for two hours to clot and centrifuged at 3000 rpm for 10 minutes. Then, each sample’s serum was isolated and poured into a microtube (17). To measure the serum levels of urea and creatinine, Auto-analyzer (BT3000, Italy) and special kits (Pars Azmoon Co., Iran) were used. After preparation and staining of glass slides, histopathological examinations were conducted and changes in the kidney tissues examined. The glass slides were histopathologically examined for tubular changes, fibrosis proportion in the space between the renal tubules, changes in Bowman’s capsule, hyalinization of renal arterioles, and expansion of mesenchymal tissue between glomerular vessels by a pathologist (18,19).

To measure kidney tissue catalase, the samples were gradually taken out of ferry and homogenized, and the amounts of their proteins and enzymes were measured (20).

### Ethical issues

Prior to the experiment, the protocols were confirmed to be in accordance with the Guidelines of Animal Ethics Committee of Shahrekord University of Medical Sciences. The experimental procedures were approved in advance by the Shahrekord University of Medical Sciences Ethics Committee (Ethics code: IR.SKUMS.REC.93-8-7).

### Statistical analysis

Data analysis was conducted by the nonparametric statistical test Kruskal-Wallis in SPSS 16 software. Moreover, median and interquartile range were used as descriptive statistics. If the variables were found to be significantly distributed among the groups according to Kruskal-Wallis test, i.e. a nonparametric analysis of variance (ANOVA) and Dunn’s test was used to make pairwise comparisons between the groups.

## Results

The results of histopathological examinations on the lams prepared from the kidney tissues are shown in Table 1. As can be seen, milder glomerular damage was seen in the group treated with 800 mg/kg of the *M. nigra* extract compared with diabetic and positive controls, and no difference in the expansion of mesenchymal tissue into renal glomerular vessels observed between the group treated with 800 mg/kg of *M. nigra* extract and diabetic and positive controls.

**Table 1:** Histopathological changes in kidney tissue in control and *Morus nigra* extract-treated groups

**Group**	**Tubular change**	**Interstitial fibrosis**	**Bowman’s capsule**	**Arteriolar hyalinosis**	**Mesangial expansion**
Normal^a^	Neg	Neg	Neg	Neg	Neg
Diab.^b^ control	Pos+	Neg	Neg	Neg	Pos/Neg
Pos.^c^ control	Pos+	Neg	Neg	Neg	Pos/Neg
*M. nigra* ^d^ 400	Pos+	Neg	Neg	Neg	Pos/Neg
*M. nigra* ^e^ 800	Pos/Neg	Neg	Neg	Neg	Neg

Abbreviatipns: Neg, negative; Pos/Neg, mild changes; Pos+, moderate changes. ^a^Normal control, healthy group; ^b^Diabetic control group; ^c^Metformin-treated (positive control), diabetic group; ^d^400 mg/kg *M. nigra* extract-treated group; ^e^800 mg/kg *M. nigra* extract-treated group.

Table 2 summarizes the changes in the serum levels of sugar, creatinine, and urea as well as kidney tissue catalase. Creatinine levels were significantly higher and urea levels significantly lower in the group treated with 800 mg/kg of *M. nigra* extract than healthy and positive control groups (*P*<0.05).

**Table 2:** Serum levels of sugar, creatinine, and urea as well as kidney tissue catalase level in the studied groups

**Groups**	**Con. Norm.** ^a^	**Con. diab.** ^b^	**Diab. Pos.** ^c^	***M. nigra*** ^d^ ** 400**	***M. nigra*** ^e^ ** 800**
Glucose (mg/dL)	103 (96-118)	362 (360-373)	336.5 (320.8-341.8)	372g^g^ (359-408)	199^h^ (185.3-210.5)
Creatinine (mg/dL)	0.5 (0.4-0.6)	0.7 (0.58-0.77)	0.5 (0.4-0.55)	0.5 (0.43-0.53)	0.8 (0.62-1.22)^gi^
Urea (mg/dL)	46 (43-53)	123 (116-125)	140.5 (111.5-163)	102.1 (99.4-104.8)	98.5^i^ (92.9-107.8)
Kid. Cat^f^(U/mg)	4.4 (4.2-4.6)	3.9 (3.7-4.2)	4.1 (3.9-4.3)	4.2 (4-4.3)	4.2 (3.8-4.6)

^a^Normal control, healthy group; ^b^Diabetic control group; ^c^Metformin-treated (positive control), diabetic group; ^d^400 mg/kg *M. nigra* extract-treated group; ^e^800 mg/kg *M. nigra* extract-treated group. ^f^Kidney catalase; ^g^Significant difference from healthy controls; ^h^Significant difference from diabetic controls; ^i^Significant difference from positive controls (*P*<0.05).

## Discussion

This study was conducted to investigate the biochemical and histopathological changes in the serum and kidneys of diabetic rats treated with hydroalcoholic *M. nigra* extract. Diabetic nephropathy is one of the most common complications due to diabetes (21,22). In this study, after injection with alloxan and induction of diabetes in the rats within two months, morphological changes were seen in their kidney tissues. These changes could be seen in microscopic examinations in all diabetized groups, but tubular changes in renal tubules were found to be mild in the group treated with 800 mg/kg *M. nigra* fruit extract.

The findings of this study are consistent with the findings of Amouoghli-Tabrizi and Mohajeri on serum urea level and kidney tissue catalase level in a study on the effect of hydroalcoholic *Brassica rapa* root extract on premature diabetic nephropathy in rats (23). In the current study, use of *M. nigra* fruit extract was found to cause mild changes compared with the changes in the diabetic control group. The study of Vessal et al on quercetin antidiabetic effects in rats demonstrated that no significant pathological changes in renal and glomerular tubules were seen in quercetin-treated rats compared with control groups (24). This inconsistency in the findings can be due to the length of the treatments (10 days in the study of Vessal et al study and 2 months in the present study).

In the present study, tubular changes, fibrosis rate in the space between the renal tubules, changes in Bowman’s capsule, hyalinization of renal arterioles, and expansion of mesenchymal tissue between glomerular vessels were histopathologically investigated. In the extract-treated groups, except for the tubular changes that were found to be mild, other variables were similar to those of the kidney tissues in the healthy controls. This finding is partly consistent with the study of Rashaki et al that investigated the preventive and therapeutic role of *Allium sativum* spray in diabetes mellitus-induced kidney tissue damage in rats diabetized with streptozotocin. The study of Rashaki et al demonstrated that diabetes caused radical increase in basement membrane thickness, proximal tubular necrosis, distal and collecting ducts, interstitial space between the tubules, atrophy of distal tubular epithelial cells, dilation of the tubules and glomeruli, mesenchymal and nodular sclerosis, loss of endocytic vesicles as well as presence of cast in the tubules and collagen in the proximal and glomerular tubules. As well, *A. sativum*-receiving group displayed decrease in tubular dilation and glomerular hypertrophy (25).

This inconsistency in the findings can be attributed to the method of inducing diabetes in the animals, the research purposes of the two studies, and differences in the phases and length of the treatments between the two studies. In this study, the expected histopathological changes in the diabetic rats’ kidneys were temporarily observed, which is due to short duration of the treatment and induction of diabetes in the studied animals. Therefore, adoption of longer durations of treatments and use of animals before induction of diabetes in future studies are recommended to arrive at more definite conclusions and obtain more generalizable evidence. In the current study, the kidney tissue catalase level was not significantly different between the healthy controls and other groups. However, this difference was found to be more marked between the healthy and diabetic controls and less marked between the extract-treated groups and the healthy controls, which represents the protective effect of *M. nigra* extract on the kidney tissue.

This study’s findings are consistent with the findings of Eskandari et al on kidney tissue catalase levels in a study on the effect of aqueous extract from rhizome of *Cynodon dactylon* L. Pers on catalase activity in rats diabetized with streptozotocin. In both studies, kidney tissue catalase level increased in the diabetized rats compared to the diabetized controls with a significant difference between diabetic controls and 500 mg/kg *C. dactylon* extract-treated group in the study of Eskandari et al (26). The minimal inconsistency in the findings of the two studies can be explained by the methods of preparing and the types of the studied extracts. The highest phenolic and flavonoid contents were reported to exist in *M. nigra* (27). These compounds, as antioxidants, can prevent many complications due to diabetes including nephropathy (25). It seems that mild changes in the kidney tissues in this study are due to *M. nigra’s* antioxidant compounds.

The average number of nephrons is approximately one million per human’s kidney. Bowman’s capsule and glomerular vessels represent a main part of the nephron and are considered to be the sites of starting the process of producing urine and excreting waste materials from the blood, any damage to which may cause several risks and complications in patients with diabetes (19). Given that nephropathy is one of the most important diabetes-induced complications (25), mesangial area expansion was histopathologically examined in this study and found to be similar in the healthy controls and 800 mg/kg *M. nigra* extract-treated group, and to undergo mild changes in 400 mg/kg *M. nigra* extract-treated group. These findings are consistent with the study of Pouladvand et al on the effects of bitter melon different doses on tissue changes (28,29).

## Conclusion

Overall, regarding the findings, 800 mg/kg *M. nigra* extract exerted more potent ameliorative effects on tubular and mesangial area changes. Therefore, adoption of longer durations of treatments after induction of diabetes and use of larger sample size are recommended to arrive at more definite conclusions regarding *M. nigra* extract and prevent diabetes-induced renal complications (30-32).

## Acknowledgments

Hereby, we gratefully thank the honorable staff of the Medical Plants Research Center, the Research and Technology Deputy of the Shahrekord University of Medical Sciences for funding this study and the faculty members of the Department of Pathology of the Isfahan University of Medical Sciences for their advice.

## Authors’ contribution

MRK and MRM conducted the research. AN studied the histopathology of kidneys’ rats. MRK designed and supervised the study. EH supervised the biochemistry tests. MRM prepared the primary draft and supervised the analysis of data. MRK edited the final draft. All authors read and signed the final manuscript.

## Conflicts of interest

The authors declared no competing interests.

## Ethical considerations

Ethical issues (including plagiarism, data fabrication, double publication) have been completely observed by the authors.

## Funding/Support

This research was supported from Shahrekord University of Medical Sciences (Grant # 1712).
